# *Takifugu obscurus *is a euryhaline fugu species very close to *Takifugu rubripes *and suitable for studying osmoregulation

**DOI:** 10.1186/1472-6793-5-18

**Published:** 2005-12-20

**Authors:** Akira Kato, Hiroyuki Doi, Tsutomu Nakada, Harumi Sakai, Shigehisa Hirose

**Affiliations:** 1 Department of Biological Sciences, Tokyo Institute of Technology, Yokohama, Japan; 2 Shimonoseki Marine Science Museum "Kaikyokan", Shimonoseki Academy of Marine Science, Shimonoseki, Japan; 3 Department of Applied Aquabiology, National Fisheries University, Shimonoseki, Japan

## Abstract

**Background:**

The genome sequence of the pufferfish *Takifugu rubripes *is an enormously useful tool in the molecular physiology of fish. Euryhaline fish that can survive both in freshwater (FW) and seawater (SW) are also very useful for studying fish physiology, especially osmoregulation. Recently we learned that there is a pufferfish, *Takifugu obscurus*, common name "mefugu" that migrates into FW to spawn. If *T. obscurus *is indeed a euryhaline fish and shares a high sequence homology with *T. rubripes*, it will become a superior animal model for studying the mechanism of osmoregulation. We have therefore determined its euryhalinity and phylogenetic relationship to the members of the *Takifugu *family.

**Results:**

The following six *Takifugu *species were used for the analyses: *T. obscurus*, *T. rubripes*, *T. niphobles*, *T. pardalis*, *T. poecilonotus*, and *T. porphyreus*. When transferred to FW, only *T. obscurus *could survive while the others could not survive more than ten days in FW. During this course of FW adaptation, serum Na^+ ^concentration of *T. obscurus *decreased only slightly, but a rapid and large decrease occurred even in the case of *T. niphobles*, a peripheral fresh water species that is often seen in brackish river mouths. Phylogenetic analysis using nucleotide sequences of the mitochondrial 16S ribosomal RNA gene of each species indicated that the six *Takifugu *species are very closely related with each other.

**Conclusion:**

*T. obscurus *is capable of adapting to both FW and SW. Its genomic sequence shares a very high homology with those of the other *Takifugu *species such that the existing *Takifugu *genomic information resources can be utilized. These properties make "mefugu", which has drawn little attention from animal physiologists until this study, a useful model animal for studying the molecular mechanism of maintaining body fluid homeostasis.

## Background

Maintenance of a stable internal environment is important for vertebrate animals to survive in a variety of habitats. Even small changes in ionic balance, osmolarity, and pH of body fluid seriously affect the survival of the animals. Strategies for maintaining body fluid homeostasis are different depending on animals and their habitats. Freshwater (FW) teleosts (modern bony fish) maintain the osmolarity of extracellular fluid around 300 mOsM, while the osmolarity of the environmental freshwater is generally less than 10 mOsM. In order to balance passive loss of salts and gain of water, they take up salts from FW through the gills and excrete a lot of dilute urine from which most of the salts have been reabsorbed by the kidney [1]. Marine teleosts also maintain the osmolarity of extracellular fluid to a level similar to that of freshwater fish, despite that the osmolarity of seawater (SW) is approx. 1000 mOsM. In order to balance passive loss of water and gain of salts, they drink seawater, absorb salts and water both in the intestine, and excrete salts through the gills [1]. The systems used by teleosts to adapt to FW and SW differ not only in the direction of ion and water movements but also in their molecular components. Euryhaline fish adapts to both FW and SW by switching these systems.

To identify the molecular components involved in body fluid homeostasis, the change of expression of each gene during adaptation of euryhaline fishes to different salinities is a potential useful marker since the genes involved are expected to be drastically up- or down-regulated during the adaptation. In fact, several genes have been identified by this strategy using euryhaline fishes such as tilapia [2], salmon [3-5], killifish [6], and eel [7-10]. However, this systematic approach is very laborious because genome sequences are not available for the euryhaline fishes that are currently being used for molecular physiological studies.

*Takifugu *is a genus of puffer fish and belongs to the family Tetradontidae of teleost fish. It consists of approx. 20 species living in the Northwest Pacific Ocean around China, Korea, and Japan [11,12]. *Takifugu *species are famous for their puffing behavior, powerful toxins in the internal organs, and edible muscle. Two species are farmed on a commercial scale: *T. rubripes *is farmed in Japan and *T. obscurus*, in Korea and China. The *Takifugu *species have an advantage as animal model, in that they have a short genome (~400 Mb) compared to those of other vertebrates including *Homo sapiens *(human, 3000 Mb), *Mus musculus *(mouse, 3000 Mb), *Gallus gallus *(chicken, 1200 Mb), *Xenopus laevis *(African clawed frog, 3100 Mb), *Danio rerio *(zebrafish, 1700 Mb), and *Oryzias latipes *(medaka, 1100 Mb) [13]. In 2002, the genome project of *T. rubripes *was completed and the sequence information is now available for free on the websites [14].

**Figure 1:**
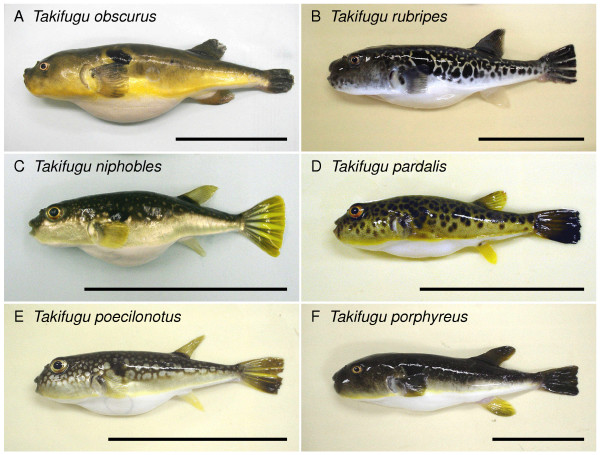
**Six *Takifugu *species used in this study**. **A**. *T. obscurus*. **B**. *T. rubripes*. **C**. *T. niphobles*. **D**. *T. pardalis*. **E**. *T. poecilonotus*. **F**. *T. porphyreus*. Scale bars represent 50 mm.

Within the genus *Takifugu*, two species are known to be anadromous, namely, *T. obscurus *[15-19] and *T. ocellatus *[16]. *T. obscurus *(Figure 1, Table 1) is found in the East China Sea, the South China Sea, and inland waters in China and the Korean Peninsula. It lives in the bottom layer of inshore and inland waters, and grows 20–40 cm in length. Most of the growth takes place in the sea but they spawn in brackish and fresh water. During the spawning season, which is from late spring to early summer, the sexually mature fish run into river estuaries and spawn in inland waters including rivers, lakes, and ponds. The fingerlings grow in the inland water and either return to the sea the next spring or they there for a few months before returning to the sea. In the sea they grow to sexually mature fish over several years, and then return to the inland water again to spawn. *T. ocellatus *is also found in an area similar to that of *T. obscurus*. *T. ocellatus *is a small species and grows to around 15 cm in length. The life cycle of *T. ocellatus *has not been well described but is expected to be similar to that of *T. obscurus*.

In this study, we focus on the suitability of *T. obscurus *as a novel animal model for studying the molecular mechanism of body fluid homeostasis. First we compared the adaptability of *T. obscurus *to FW with those of other *Takifugu *species, and showed that only *T. obscurus *is fully adaptable to both SW and FW. Next we demonstrated that changes in blood Na^+ ^concentration of *T. obscurus *during FW adaptation are kept within the physiological range while those of *T. niphobles *decline beyond the range. Finally we isolated and sequenced 16S ribosomal genes from six *Takifugu *species including *T. obscurus*, *T. niphobles*, and *T. rubripes*, and demonstrated that those sequences are 99% identical within the genus *Takifugu*. With the euryhalinity and applicability of the currently available fugu genome sequence, we conclude that *T. obscurus *is a useful animal model for studying the mechanism of osmoregulation.

## Results

### Survival of *Takifugu *species in FW

**Figure 2:**
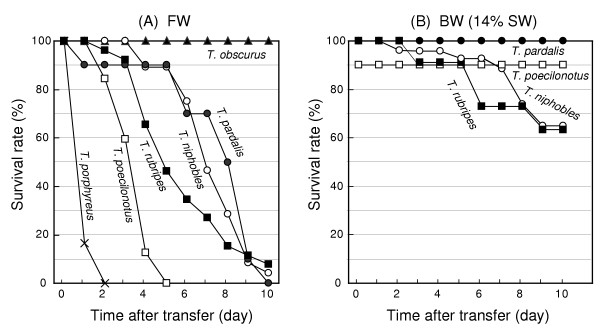
**Survival rates of the *Takifugu *species after a direct transfer from seawater (SW) to freshwater (FW) or brackish water (BW)**. Fourteen-percent SW was used as BW. Numbers of fishes used for the analyses were: *T. obscurus*, n = 18 for FW; *T. niphobles*, n = 35 for FW and n = 36 for BW; *T. pardalis*, n = 10 for FW and n = 6 for BW; *T. poecilonotus*, n = 32 for FW and n = 10 for BW; *T. rubripes*, n = 26 for FW and n = 11 for BW; and *T. porphyreus*, n = 6 for FW.

A summary on six *Takifugu *species used in this study is shown in Figure 1 and Table 1. The survival rate of each species after transfering from SW to FW is shown in Figure 2A. The results show the mean values of several experiments. The mean survival in FW was: 1.2 ± 0.2 days, 3.6 ± 0.2 days, 5.5 ± 0.4 days, 7.0 ± 0.3, 7.5 ± 0.8 days, and more than 10 days for *T. porphyreus*, *T. poecilonotus*, *T. rubripes*, *T. niphobles*, *T. pardalis*, and *T. obscurus*, respectively. In a separate experiment, we confirmed that *T. obscurus *could survive for at least 3 weeks in FW without any apparent difficulties (data not shown). These data suggest that only *T. obscurus *is fully adaptable to both FW and SW among the six *Takifugu *species tested. Of the five species that could not survive in FW, four (*T. niphobles, T. rubripes, T. pardalis *and *T. poecilonotus*) were able to adapt to BW (14% SW) (Figure 2B).

The fishes that survived for 10 days in BW were transferred to FW and survival was monitored (time course data not shown). Mean survival in FW were: 3.1 ± 0.6 days, 4.6 ± 0.6 days, and 5.5 ± 0.7 days for *T. niphobles *(n = 7), *T. poecilonotus *(n = 8), and *T. pardalis *(n = 6), respectively. Mean survival in FW following the transfer from BW did not differ significantly between *T. poecilonotus *and *T. pardalis*, and was short for *T. niphobles *(P < 0.001) when compared to the survival of those that were transferred from SW to FW. These results indicate that 10 days' adaptation to BW does not improve the adaptability of *T. poecilonotus*, *T. pardalis*, and *T. niphobles *to FW.

### Changes in serum osmolarity and concentrations of ions and urea during adaptation

To gain insights into the way that the *Takifugu *species adapt to different salinities, we sampled the blood from two species, *T. obscurus *and *T. niphobles*, and determined serum osmolarity and concentrations of ions and urea (Table 2). In SW, serum osmolarity and ion concentration of *T. obscurus *and *T. niphobles *were similar to those reported for other teleost fish [1]. When transferred to FW, however, significant changes were observed in serum osmolarity and concentrations of Na^+ ^and Cl^- ^for *T. niphobles*, whereas the changes were small for *T. obscurus*. The reductions in osmolarity during FW adaptation of *T. obscurus *and *T. niphobles *were -17 and -148 mOsM, respectively. The decrements of serum concentrations of Na^+ ^and Cl^- ^during FW adaptation were -13 and -16 mM in *T. obscurus*, and -69 and -52 mM in *T. niphobles*, respectively. These results suggest that *T. obscurus *has a much stronger ability to maintain body fluid homeostasis against salinity fluctuations and can survive in FW.

**Figure 3:**
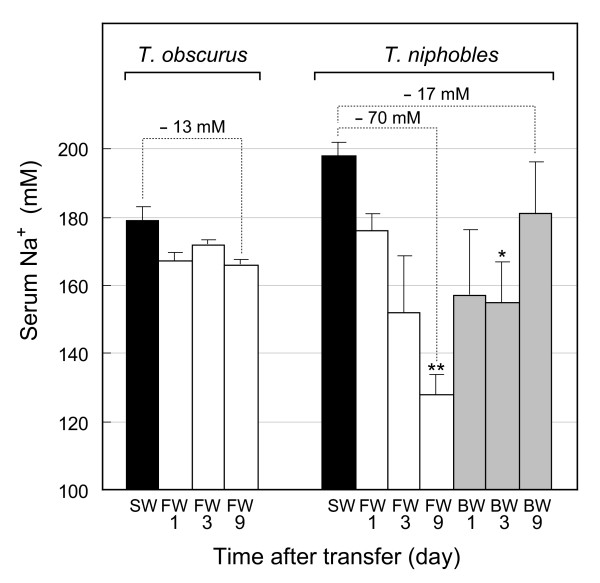
**Changes in serum Na^+ ^concentration during transfer from seawater (SW) to freshwater (FW) or to brackish water (BW)**. Results of *T. obscurus *and *T. niphobles *are shown on the left and right, respectively. Serum Na^+ ^concentrations of the fish that adapted to SW, FW, and BW are shown as the black, white, and gray bars, respectively (n = 3–4). 14% SW was used as BW. *P < 0.01, **P < 0.001.

Figure 3 shows the time course of changes in serum Na^+ ^concentration following exposure of *T. obscurus *and *T. niphobles *to low salinities. In the case of *T. obscurus*, a slightly decreased level that was observed on day 1, remained throughout the course, but in the case of *T. niphobles*, a relatively large decrease occurred continuously until death. In BW where *T. niphobles *exhibited 64% survival rate (Figure 2), a significant recovery of the decreased serum Na^+ ^levels was observed on day 9 (Figure 3). The standard errors of serum Na^+ ^concentration of *T. niphobles *(7.6–32 mM) were much larger than those of *T. obscurus *(2.3–7.9 mM), suggesting that the individual differences of adaptability to FW and BW are large in *T. niphobles*. In *T. niphobles *the decrease in serum Cl^- ^was more extensive than that in serum Na^+^. In *T. obscurus *serum Cl^- ^decreased while Na^+ ^and osmolarity remained unchanged.

### Comparison of nephron structure of *Takifugu *species

**Figure 4:**
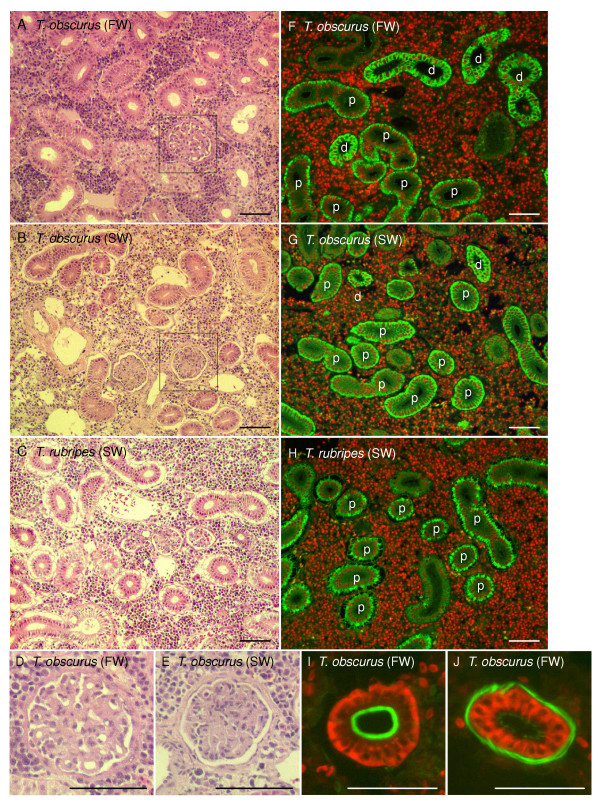
**Renal structure of *Takfiugu *kidneys**. **A–E**. Paraffin-embedded sections of the kidneys of indicated *Takifugu *species were stained with hematoxylin and eosin and examined for abundance of glomeruli. All the other species, *T. niphobles*, *T. pardalis*, *T. poecilonotus*, and *T. porphyreus*, also have glomerulous nephron (data not shown). **D**. Higher magnification view of the glomeruli of FW-acclimated *T. obscurus *indicated by a box in A. **E**. Higher magnification views of the glomeruli of SW-acclimated *T. obscurus *indicated by boxes in B. **F–H**. Paraffin-embedded sections of the kidneys of indicated *Takifugu *species were stained with anti-Na^+^-K^+^-ATPase (NKA) antibody (green) and Hoechst 33342 (red). NKA antibody strongly stained basolateral surface of proximal segment (p) and entire cell of distal segment (d). *T. niphobles*, *T. pardalis*, and *T. poecilonotus *showed similar result to *T. rubripes *(data not shown). **I–J**. Frozen sections of the kidneys of *T. obscurus *were stained with anti-NKA antibody (red) and Alaxa Fluor 488-labeled phalloidin (green). Phalloidin binds to actin filaments, and strongly stains a well-developed apical brush border of proximal segments. **I**. Proximal segment of the nephron of *T. obscurus*. **J**. Distal segment of the nephron of *T. obscurus*. All scale bars represent 50 μm.

Kidney sections of the six *Takifugu *species were analyzed to compare their nephron structures. Under light microscope, a number of glomeruli were observed within all sections stained with hematoxylin-eosin, demonstrating that all six *Takifugu *species have glomerular nephrons (Figure 4A–C). The glomeruli of FW-acclimated *T. obscurus *appeared to be loose compared to those of the SW-acclimated fish (Figure 4D–E). There was no clear difference between those species rich in glomeruli at the histological level.

To characterize the segments of the renal tubules, kidney sections of *T. obscurus, T. rubripes*, *T. niphobles*, *T. pardalis*, and *T. poecilonotus *were stained with anti-Na^+^-K^+^-ATPase (NKA) antibody, and observed under a fluorescence microscope. NKA is the most important molecule that provides a driving force for many transporting systems in the renal tubules, and the patterns of NKA localization are different among the segments of renal tubule (Figure 4I–J) [20]. In both FW- and SW-acclimated *T. obscurus*, proximal and distal segments were clearly observed (Figure 4F–G). In contrast, the distal segment is not found in *T. rubripes*, *T. niphobles*, *T. pardalis*, and *T. poecilonotus *(Figure 4H).

### Phylogeny of *Takifugu *species

**Figure 5:**
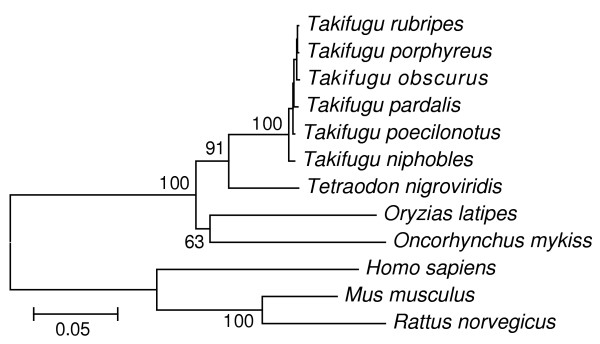
**Phylogenetic relationship between *Takifugu *and other species**. 1.1-kb nucleotide sequences of the mitochondrial 16S ribosomal RNA gene of each species were used for the analyses. Bootstrap values from 2,000 times replications are indicated at major nodes. Bars indicate 5% replacement of a nucleotide per site. Accession numbers were as follows: *T. niphobles*, AB199318; *T. poecilonotus*, AB199319; *T. pardalis*, AB199320; *T. rubripes*, AB199321; *T. obscurus*, AB199322; *T. porphyreus*, AB199323; *T. nigroviridis*, CR688806; *O. latipes*, NC_004387; *O. mykiss*, NC_001717; *H. sapiens*, J01415; *M. musculus*, J01420; and *R. norvegicus*, X14848.

To know the phylogenetic relationship of the *Takifugu *species, we isolated the mitochondrial 16S rRNA gene from each species and determined the sequence. Resulting data were compared with the sequences of the 16S rRNA genes of other species in databases, and a phylogenetic tree was constructed (Figure 5). Surprisingly, the *Takifugu *species were very closely related each other. The identities of 16S rRNA within the *Takifugu *species are 99% whereas those between *Takifugu *and *Tetraodon nigroviridi*, *Oryzias latipes*, or *Homo sapiens *were 86%, 77%, and 63%, respectively. Our preliminary results of the nucleotide sequences of several cDNA clones for ion transporters (Na^+^/H^+ ^exchangers; accession numbers AB200326–AB200333) and hormone receptors (members of the adrenomedullin receptor family: accession numbers AB219765–AB219771, AB219835–AB219840) [21] of *T. obscurus *were 99% identical to those of *T. rubripes *including the non-coding sequences (data not shown). These results suggest that the *Takifugu *species diversified very recently and the genome resources of *T. rubripes *can be used for studying the *T. obscurus *genes and their products.

## Discussion

Through analyses of the ability of the *Takifugu *species to adapt to FW, we have demonstrated that only *T. obscurus *exhibits a high adaptability to both FW and SW. This observation is consistent with their natural anadromous habitats (Table 1). In our analyses, we used sexually immature fish (~10 g) and large fish (~350 g) with well developed testis or ovary. All the *T. obscurus *survived in both FW and SW for more than 10 days and they looked healthy, suggesting that size and sexual maturation do not affect their adaptability. Recently, Yan *et al*. reported the effect of salinity on food intake, growth, and survival of *T. obscurus *(~45 g) [19]. They cultured *T. obscurus *in FW, BW, and SW for 54 days and compare their level of food-intake and growth rates. The fish survived and grew under all conditions tested, and the growth rates in low-salinity BW (23% SW) were better than those in FW, SW and high-salinity BW (51% SW). Their observation demonstrated that *T. obscurus *grows under a wide range of salinities and low-salinity BW is the best condition for young *T. obscurus *to grow.

Our analyses also demonstrated that many other *Takifugu *species exhibit a relatively high ability to cope with salinity changes. *T. niphobles*, *T. rubripes*, *T. pardalis*, and *T. poecilonotus *can survive in FW for several days and in BW for more than 10 days, suggesting that the *Takifugu *species are potentially euryhaline. These results are consistent with their natural brackish/marine habitats; they are sometimes found in brackish river mouths (Table 1). It is known that *T. rubripes *spawn in the entrance of bays. The fingerlings grow in shallow and river mouths of bays for one year, and then go to the broad ocean [22]. Han *et al*. demonstrated that the best growing salinity of *T. rubripes *weighting ~0.02, ~1.2 and ~25 g were 73–91%, 29%, and 43% SW, respectively [23]. Thus change of the environmental salinity is important for the growth of the fingerlings of *T. rubripes*.

During the acclimation to FW, serum Cl^- ^of *T. obscurus *decreased although Na^+ ^and osmolarity remained unchanged. In *T. niphobles *the decrease in serum Cl^- ^was more extensive than that in serum Na^+^. These results suggest that the mechanisms whereby Cl^- ^and Na^+ ^are regulated differ. The decrease in serum Cl^- ^during FW acclimation has also been observed in Japanese eel (*Anguilla japonica*) [24] and spotted green pufferfish (*Tetraodon nigroviridis*) [20]. In *Tetraodon *and *Takifugu *species, the other electrolytes that compensate for Cl^- ^were not determined. In the case of Japanese eel, serum SO_4_^2- ^concentration increases from ~1 to ~19 mM during acclimation from SW to FW [24]. The expressions of kidney sulfate transporters are drastically induced during FW acclimation, suggesting that the serum SO_4_^2- ^reabsorbed by the kidney compensates for Cl^- ^and helps improve the survival of eel in FW [24].

Some reports have categorized pufferfish as aglomerular [25,26]. However, glomerular nephrons was observed in the species of from four genuses of the Tetraodontidae family, namely, *Canthigaster rivulatus *[27], *Tetraodon nigroviridis *[20], *Sphoeroides testudineus *[28], two *Takifugu *species reported by Ogawa [27], and six *Takifugu *species in this study (Figure 4). We think that many of the Tetraodontidae species are glomerular. The increase in size of the glomerulus after transferring to FW (Figure 4D–E) was also found in the threespine stickleback (*Gasterosteus aculeatus *L.) [29]. In general, the largest difference between FW fish and glomerular SW fish regarding structure of the renal tubules is the presence or absence of a distal segment, which acts as a urine-diluting segment in FW fish [30]. Most of the euryhaline fish have a FW-fish type of nephron such as the European eel (*Anguilla vulgaris*), Pacific pink salmon (*Oncorhynchus gorbuscha*), rainbow trout (*Oncorhynchus mykiss*), southern flounder (*Paralichthys lethostigma*), armored sculpin (*Leptocottus armatus*), medaka (*Oryzias latipes*), and spotted green pufferfish [20,30]. In the *Takifugu *species, we demonstrated that only mefugu (*T. obscurus*) has the FW-fish type of nephron with a distal segment, and the other species have a SW-fish type of nephron lacking a distal segment (Figure 4F–J). These results are completely consistent with the ability of those species to adapt to FW, thus the presence of a distal segment is one of the most important factors that allow *T. obscurus *to be highly adaptalbe to a wide range of salinities.

*Tetraodon nigroviridis *(spotted green pufferfish) is a small pufferfish less than 10 cm in length that lives in brackish river and estuaries of Southeast Asia. *T. nigroviridis *also has a compact genome like the *Takifugu *species, and the whole genome was sequenced in 2004 [31]. Recently, Lin *et al*. have demonstrated the strong adaptability of *T. nigroviridis *to FW, BW, and SW and its use in studies on osmoregulation [20]. We think that both *T. nigroviridis *and *T. obscurus *are good models for studying osmoregulation. The advantage with *T. nigroviridis *is that it is readily available. The advantage with *T. obscurus *is that it can be used in a wide range of size (2–20 cm) and compare the functions of the gill and kidney with those of other *Takifugu *species that can not adapt to FW.

Many molecules have been identified as components of the chloride cells (or mitochondria-rich cells), the major site of ion regulation in the gill: transporters, channels, and pumps for Na^+^, K^+^, Cl^-^, HCO_3_^-^, H^+^, Ca^2+^, water, and urea; carbonic anhydrase; and hormone receptors [32]. However, the complete physiological function of the chloride cells cannot be explaned by those components alone, and identification of further players is necessary. Furthermore, little is known of the molecular biology of osmoregulation by the kidney and intestine of teleost fish: NKA [20], sulphate transporters [24], urea transporter [33], chloride channel [34], Ca^2+^-sensing receptor [35], V-type H^+^-ATPases [36] in the kidney; Na-Pi cotransporter [37] and aquaporin water channels in both the kidney and intestine [38-40]; and Na^+^/K^+^/2Cl^- ^cotransporter in the stomach and intestine [41]. By determining the differences in gene expression patterns in the gill, intestine, and kidney of FW- and SW-acclimated mefugu (*T. obscurus*), we would be able to identify the genes that are important for osmoregulatory adaptation.

## Conclusion

• Mefugu (*T. obscurus*) is an anadromous fish of the genus *Takifugu *that has a strong ability to maintain body fluid homeostasis during adaptation to low and high environmental salinities and is fully adaptable to both FW and SW.

• Members of the genus *Takifugu *are very closely related and share ~99% sequence identities in their genomes as shown by a phylogenetic analysis using the mitochondrial DNA sequence for the 16S ribosomal RNA gene.

• The nephrons of FW- or SW-acclimated *T. obscurus *exhibit a structure that is typical of FW fish. On the other hand, *T. rubripes*, *T. niphobles*, *T. pardalis*, *T. poecilonotus*, and *T. porphyreus *have nephrons of that are typical of SW glomerular fish.

• *T. obscurus *can be used as an animal model for studying the molecular mechanism of osmoregulation by exploiting the *Takifugu *genome resources.

## Methods

### Animals and transfer experiment

The animal protocols and procedures were approved by the Institutional Animal Care and Use Committee of Tokyo Institute of Technology and conformed to the American Physiological Society's *Guiding Principles in the Care and Use of Laboratory Animals*. *T. obscurus *(10–350 g) were cultured in a brackish river in Korea and China. The fish cultured in BW (14% SW) were transported to The Shimonoseki Marine Science Museum in Japan and kept in 150–2000-l tanks containing BW. The fish were then acclimated to SW for 7–14 days. None of the fish died during the acclimation to SW. To determe FW adaptability, the SW in the tank was gradually replaced with FW by pouring FW at a speed that allowed a complete replacement after 1–2 h. Some fish were transferred to FW directly. Survival was then monitored every 12 h for 10 days.

Other species were caught or cultured in seawater. *T. rubripes *(30–4200 g) were cultured and sampled at the Japan Sea. *T. niphobles *(18–128 g), *T. pardalis *(29–175 g), *T. poecilonotus *(18–43 g), and *T. porphyreus *(521–1000 g) were sampled at the Japan Sea. They were transported to the Aquarium and kept in 200–5700-l tanks containing SW. Their adaptability to FW and BW were determined as described above.

All fish used in the analyses were adult fish. The normal size of each species is shown in Table I. Most of the *T. niphobles*, *T. pardalis*, *T. poecilonotus*, and *T. porphyreus *were sexually mature adult fish. *T. obscurus *and *T. rubripes *were mixtures of mature and immature fish. The distinction between the species was performed according to Nakabo [11].

### Blood analyses

*T. obscurus *and *T. niphobles *were maintained in SW and transferred to FW or BW (14% SW). Bloods were collected from the fish in SW and those in FW or BW after 1, 3, and 9 days of the transfer. Healthy fish that had adapted to in various conditions were anaesthetized by immersion in 0.1% ethyl *m*-aminobenzoate methanesulfonate, and blood was collected from the hepatic vein or heart. Serum from *T. obscurus *and *T. niphobles *were diluted in water at the ratio of 1:2 and 1:8, respectively, and used for the analyses. Serum osmolarity was measured by a cryoscopic method. Concentrations of Na^+^, K^+^, and Cl^- ^were measured by the established electrode methods. Ca^2+ ^and Mg^2+ ^concentrations were determined by the *o*-cresolphthalein complexone method and xylysine blue method, respectively. Urea nitrogen concentration in the serum was measured by standard urease assay. The dilution of serum in water did not affect the results (data not shown). These measurements were conducted by SRL Laboratories (Tokyo, Japan).

### Histochemistry

Kidneys of pufferfish were fixed in 4% paraformaldehyde in 0.1 M phosphate buffer, pH 7.4, embedded in paraffin, sectioned, and stained with hematoxylin and eosin according to standard procedures.

For the immunohisotchemical analyses, the kidneys were fixed in 4% paraformaldehyde in 0.1 M phosphate buffer, pH 7.4, at 4°C for 2 h, and rinsed in phosphate-buffered saline (PBS: 137 mM NaCl, 2.7 mM KCl, 6.5 mM Na_2_HPO_4_, 1.5 mM KH_2_PO_4_, pH 7.4) containing 10% (w/v) sucrose. The fixed tissues were cryoprotected through a range of increasing sucrose concentrations up to 20%, quick frozen in an optimum cutting temperature compound (Tissue Tek).

The frozen sections (6 μm) and paraffin-embedded sections (6 μm) were prepared, permeabilized with 0.1% Triton X-100 in PBS at 20°C for 10 min, incubated with 5% fetal bovine serum (FBS) in PBS at 20°C for 1 h, and incubated with anti-Na^+^-K^+^-ATPase rabbit antiserum [8] (1:1,000) in PBS containing 5% FBS at 20°C for 8 h. After washing with PBS, the sections were incubated with a mixture of Alexa Fluor-488- or Alexa Fluor-546-labeled secondary antibody (Molecular Probes; 1:2,000 dilution), Alexa Fluor-488-labeled phalloidin (Molecular Probes; 0.15 μM), and Hoechst 33342 (Molecular Probes; 100 ng/ml) in PBS containing 5% FBS at 20°C for 1 h. The sections were mounted on antifade glycerol (90% glycerol, 10% 10 × PBS, and 0.1% 1,4-phenylenediamine, pH 7.4). Fluorescence was detected using a fluorescence microscope (Carl Zeiss). The images were obtained with a high-resolution digital charge-coupled device (CCD) camera (AxioCam HRm, Carl Zeiss) and processed with an AxioVision 4.1 software (Carl Zeiss).

The following pufferfish were used for the analyses: *T. obscurus *acclimated to FW for 9 days; *T. obscurus *acclimated to SW for 9 days; *T. rubripes*, *T. niphobles*, *T. pardalis*, *T. poecilonotus*, and *T. porphyreus *maintained in SW.

### Phylogenetic analyses

Mitochondrial DNA from *T. obscurus*, *T. rubripes*, *T. niphobles*, *T. pardalis*, *T. poecilonotus*, and *T. porphyreus *was extracted from the fin, and used for isolation of genes for 16S rRNA by PCR as described elsewhere [42]. Two sets of primers were used: L1854 (5'-AAACCTCGTACCTTTTGCAT-3') and H2582 (5'-ATTGCGCTACCTTTGCACGGT-3') for amplification of the anterior half of the 16S rRNA genes, and L2503 (5'-CACAAGCCTCGCCTGTTTACCA-3') and H3058 (5'-TCCGGTCTGAACTCAGATCACGTA-3') for the amplification of the posterior half. Products of PCR were purified and directly sequenced by the dideoxy chain termination method with an automated DNA sequencer (Model 310; Applied Biosystems, Foster City, CA). The GenBank accession number for the sequence of each gene is as follows: *T. niphobles*, AB199318; *T. poecilonotus*, AB199319; *T. pardalis*, AB199320; *T. rubripes*, AB199321; *T. obscurus*, AB199322; and *T. porphyreus*, AB199323.

For the evolutionary analyses, the nucleotide sequences were aligned using Clustal W software [43], and then a phylogenetic tree was constructed by the neighbor-joining method [44] using MEGA software [45] based on Jukes-Cantor evolutionary distances [44]. Statistical analysis was performed by bootstrap methods [44].

## List of abbreviations

SW – seawater, FW – freshwater, BW – brackish water

## Authors' contributions

AK and SH planned of and designed the study, and wrote the manuscript. HD planned the sections of the study, and performed the operations relating to the supply, transfer, and maintenance of the fish. HD and AK performed the salinity transfer analyses. HS cloned and sequenced genes for 16S rRNA, and collected information on ecobiology of the *Takifugu *species. AK performed blood assays and construction of the phylogenetic tree. TN performed the histochemical analyses. All authors read and approved the final manuscript.

## Figures and Tables

**Table 1:** List of *Takifugu *species used in this study.

Adaptability	Species (Common name)	Environment (Total length)	Spawning	Region	Ref.
Seawater fish, Anadromous freshwater fish	*Takifugu obscurus *(Obscure puffer, Mefugu)	Sea River 20–40 cm	River	East Asia	[12,15-17,46]
Seawater fish, Peripheral freshwater fish	*Takifugu niphobles *(Grass puffer, Kusafugu)	Seacoast, River mouth* 15 cm	Sea	East Asia	[12,15,47]
Seawater fish	*Takifugu pardalis *(Panther puffer, Higanfugu)	Sea, River mouth** 20–38 cm	Sea	East Asia	[12,22]
	*Takifugu poecilonotus *(Fine-patterned puffer, Komonfugu)	Sea, River mouth*** 20 cm	Sea	East Asia	[12,15]
	*Takifugu rubripes *(Ocellate puffer, Tiger puffer, Torafugu)	Sea, River mouth*** 35–80 cm	Sea	East Asia	[12,15,22]
	*Takifugu porphyreus *(Genuin puffer, Purple puffer, Mafugu)	Sea 20–50 cm	Sea	East Asia	[12,22]

* Adult fish are often seen in BW river mouths and sometimes seen in FW rivers.

** Adult fish are sometimes seen in BW river mouths [48].

*** Fingerlings are often seen in brackish river mouths [22].

**Table 2:** Serum osmolarity (mOsM) and concentration (mM) of ions and urea

Species	Condition	mOsM	Na^+^	K^+^	Ca^2+^	Mg^2+^	Cl^-^	Urea
*T. obscurus*	SW	363 ± 9.4	179 ± 3.9	2.8 ± 0.4	3.1 ± 0.2	1.8 ± 0.1	144 ± 3.3	2.6 ± 0.4
	FW	346 ± 5.7	166 ± 1.2	3.0 ± 0.7	3.5 ± 0.3	1.3 ± 0.1	128 ± 2.1*	3.7 ± 0.4
*T. niphobles*	SW	392 ± 5.6	197 ± 3.8	N.D.	2.8 ± 0.1	1.6 ± 0.1	126 ± 3.6	1.0 ± 0.4
	FW	244 ± 9.5**	128 ± 5.7**	N.D.	2.2 ± 0.2	0.9 ± 0.2	74 ± 2**	0.4 ± 0.1
	BW	355 ± 33	181 ± 15	N.D.	3.0 ± 0.3	2.0 ± 0.2	104 ± 12	0.6 ± 0.1

Values are means ± SE; n = 4. *P < 0.01, **P < 0.001.

## References

[B1] Karnaky KJ, Evans DH (1998). Osmotic and ionic regulation. The Physiology of Fishes.

[B2] Fiol DF, Kultz D (2005). Rapid hyperosmotic coinduction of two tilapia (*Oreochromis mossambicus*) transcription factors in gill cells. Proc Natl Acad Sci U S A.

[B3] Pan F, Zarate J, Bradley TM (2002). A homolog of the E3 ubiquitin ligase Rbx1 is induced during hyperosmotic stress of salmon. Am J Physiol Regul Integr Comp Physiol.

[B4] Pan F, Zarate J, Choudhury A, Rupprecht R, Bradley TM (2004). Osmotic stress of salmon stimulates upregulation of a cold inducible RNA binding protein (CIRP) similar to that of mammals and amphibians. Biochimie.

[B5] Smith TR, Tremblay GC, Bradley TM (1999). Hsp70 and a 54 kDa protein (Osp54) are induced in salmon (*Salmo salar*) in response to hyperosmotic stress. J Exp Zool.

[B6] Scott GR, Richards JG, Forbush B, Isenring P, Schulte PM (2004). Changes in gene expression in gills of the euryhaline killifish Fundulus heteroclitus after abrupt salinity transfer. Am J Physiol Cell Physiol.

[B7] Mistry AC, Kato A, Tran YH, Honda S, Tsukada T, Takei Y, Hirose S (2004). FHL5, a novel actin-binding protein, is highly expressed in eel gill pillar cells and responds to wall tension. Am J Physiol Regul Integr Comp Physiol.

[B8] Mistry AC, Honda S, Hirata T, Kato A, Hirose S (2001). Eel urea transporter is localized to chloride cells and is salinity dependent. Am J Physiol Regul Integr Comp Physiol.

[B9] Suzuki Y, Itakura M, Kashiwagi M, Nakamura N, Matsuki T, Sakuta H, Naito N, Takano K, Fujita T, Hirose S (1999). Identification by differential display of a hypertonicity-inducible inward rectifier potassium channel highly expressed in chloride cells. J Biol Chem.

[B10] Miyamoto K, Nakamura N, Kashiwagi M, Honda S, Kato A, Hasegawa S, Takei Y, Hirose S (2002). RING finger, B-box, and coiled-coil (RBCC) protein expression in branchial epithelial cells of Japanese eel, *Anguilla japonica*. Eur J Biochem.

[B11] Nakabo T (2002). Fishes of Japan – with pictorial keys to the species.

[B12] Masuda H, Amaoka K, Araga C, Uyeno T, Yoshino T (1984). The Fishes of the Japanese Archipelago.

[B13] Venkatesh B, Gilligan P, Brenner S (2000). Fugu: a compact vertebrate reference genome. FEBS Lett.

[B14] Aparicio S, Chapman J, Stupka E, Putnam N, Chia JM, Dehal P, Christoffels A, Rash S, Hoon S, Smit A, Gelpke MD, Roach J, Oh T, Ho IY, Wong M, Detter C, Verhoef F, Predki P, Tay A, Lucas S, Richardson P, Smith SF, Clark MS, Edwards YJ, Doggett N, Zharkikh A, Tavtigian SV, Pruss D, Barnstead M, Evans C, Baden H, Powell J, Glusman G, Rowen L, Hood L, Tan YH, Elgar G, Hawkins T, Venkatesh B, Rokhsar D, Brenner S (2002). Whole-genome shotgun assembly and analysis of the genome of *Fugu rubripes*. Science.

[B15] Kim IS (1997). Freshwater Fishes.

[B16] Wu HL, Jin XB, Ni Y (1978). Toxic and Pharmacological Fish in China.

[B17] Xu C, East China Sea Fisheries Institute, Chinese Academy of Fisheries Science (1990). *Takifugu obscurus *(Abe). The Fishes of Shanghai Area.

[B18] Yang Z, Chen YF (2004). Induced ovulation in obscure puffer *Takifugu obscurus *by injections of LHRH-a. Aquacul Int.

[B19] Yan M, Li Z, Xiong B, Zhu J (2004). Effects of salinity on food intake, growth, and survival of pufferfish (*Fugu obscurus*). J Appl Ichthyol.

[B20] Lin CH, Tsai RS, Lee TH (2004). Expression and distribution of Na, K-ATPase in gill and kidney of the spotted green pufferfish, *Tetraodon nigroviridis*, in response to salinity challenge. Comp Biochem Physiol A Mol Integr Physiol.

[B21] Nag K, Kato A, Nakada T, Hoshijima K, Mistry AC, Takei Y, Hirose S (2006). Molecular and functional characterization of adrenomedullin receptors in pufferfish. Am J Physiol Regul Integr Comp Physiol.

[B22] Fujita S (1962). Studies on life history and aquaculture of Japanese puffer fishes. Rep Nagasaki Pref Inst Fish.

[B23] Han K, Chuang H, Matsui S, Furuichi M, Kitajima C (1995). Effect of ambient salinity on growth, survival rate, and feed efficiency in the early stage of puffer fish *Takifugu rubripes*. Nippon Suisan Gakkaishi.

[B24] Nakada T, Zandi-Nejad K, Kurita Y, Kudo H, Broumand V, Kwon CY, Mercado A, Mount DB, Hirose S (2005). Roles of Slc13a1 and Slc26a1 sulfate transporters of eel kidney in sulfate homeostasis and osmoregulation in freshwater. Am J Physiol Regul Integr Comp Physiol.

[B25] Vize PD (2004). A Homeric view of kidney evolution: A reprint of H.W. Smith's classic essay with a new introduction. Anat Rec A Discov Mol Cell Evol Biol.

[B26] Edwards JG (1928). Studies on aglomerular and glomerular kidneys. Am J Anat.

[B27] Ogawa M (1962). Comparative study on the internal structure of the teleostean kidney. Sci Rept Saitama Univ.

[B28] Prodocimo V, Freire CA (2003). Glomeruli and renal tubules are restricted to the cranial kidney of the adult estuarine *Sphoeroides testudineus*. J Fish Biol.

[B29] Ogawa M (1968). Seasonal difference of glomerular change of marine form of the stickleback, *Gasterosteus aculeatus *L. after transferred into fresh water. Sci Rept Saitama Univ.

[B30] Hickman CP, Trump BF, Hoar WS, Randall DJ (1969). The Kidney. Fish Physiology.

[B31] Jaillon O, Aury JM, Brunet F, Petit JL, Stange-Thomann N, Mauceli E, Bouneau L, Fischer C, Ozouf-Costaz C, Bernot A, Nicaud S, Jaffe D, Fisher S, Lutfalla G, Dossat C, Segurens B, Dasilva C, Salanoubat M, Levy M, Boudet N, Castellano S, Anthouard V, Jubin C, Castelli V, Katinka M, Vacherie B, Biemont C, Skalli Z, Cattolico L, Poulain J, De BV, Cruaud C, Duprat S, Brottier P, Coutanceau JP, Gouzy J, Parra G, Lardier G, Chapple C, McKernan KJ, McEwan P, Bosak S, Kellis M, Volff JN, Guigo R, Zody MC, Mesirov J, Lindblad-Toh K, Birren B, Nusbaum C, Kahn D, Robinson-Rechavi M, Laudet V, Schachter V, Quetier F, Saurin W, Scarpelli C, Wincker P, Lander ES, Weissenbach J, Roest CH (2004). Genome duplication in the teleost fish *Tetraodon nigroviridis *reveals the early vertebrate proto-karyotype. Nature.

[B32] Hirose S, Kaneko T, Naito N, Takei Y (2003). Molecular biology of major components of chloride cells. Comp Biochem Physiol B Biochem Mol Biol.

[B33] Mistry AC, Chen G, Kato A, Nag K, Sands JM, Hirose S (2005). A novel type of urea transporter, UT-C, is highly expressed in proximal tubule of seawater eel kidney. Am J Physiol Renal Physiol.

[B34] Miyazaki H, Kaneko T, Uchida S, Sasaki S, Takei Y (2002). Kidney-specific chloride channel, OmClC-K, predominantly expressed in the diluting segment of freshwater-adapted tilapia kidney. Proc Natl Acad Sci U S A.

[B35] Loretz CA, Pollina C, Hyodo S, Takei Y, Chang W, Shoback D (2004). cDNA cloning and functional expression of a Ca^2+^-sensing receptor with truncated C-terminal tail from the Mozambique tilapia (*Oreochromis mossambicus*). J Biol Chem.

[B36] Perry SF, Furimsky M, Bayaa M, Georgalis T, Shahsavarani A, Nickerson JG, Moon TW (2003). Integrated responses of Na^+^/HCO_3_^- ^cotransporters and V-type H^+^-ATPases in the fish gill and kidney during respiratory acidosis. Biochim Biophys Acta.

[B37] Kohl B, Herter P, Hulseweh B, Elger M, Hentschel H, Kinne RK, Werner A (1996). Na-Pi cotransport in flounder: same transport system in kidney and intestine. Am J Physiol.

[B38] Aoki M, Kaneko T, Katoh F, Hasegawa S, Tsutsui N, Aida K (2003). Intestinal water absorption through aquaporin 1 expressed in the apical membrane of mucosal epithelial cells in seawater-adapted Japanese eel. J Exp Biol.

[B39] Martinez AS, Cutler CP, Wilson GD, Phillips C, Hazon N, Cramb G (2005). Regulation of expression of two aquaporin homologs in the intestine of the European eel: effects of seawater acclimation and cortisol treatment. Am J Physiol Regul Integr Comp Physiol.

[B40] Martinez AS, Cutler CP, Wilson GD, Phillips C, Hazon N, Cramb G (2005). Cloning and expression of three aquaporin homologues from the European eel (*Anguilla anguilla*): effects of seawater acclimation and cortisol treatment on renal expression. Biol Cell.

[B41] Cutler CP, Cramb G (2002). Two isoforms of the Na^+^/K^+^/2Cl^- ^cotransporter are expressed in the European eel (*Anguilla anguilla*). Biochim Biophys Acta.

[B42] Watanabe K, Iguchi K, Hosoya K, Nishida M (2000). Phylogenetic relationships of the Japanese minnows, Pseudorasbora (Cyprinidae), as inferred from mitochondrial 16S rRNA gene sequences. Ichthyol Res.

[B43] Thompson JD, Higgins DG, Gibson TJ (1994). CLUSTAL W: improving the sensitivity of progressive multiple sequence alignment through sequence weighting, position-specific gap penalties and weight matrix choice. Nucleic Acids Res.

[B44] Nei M, Kumar S (2000). Molecular Evolution and Phylogenetics.

[B45] Kumar S, Tamura K, Jakobsen IB, Nei M (2001). MEGA2: molecular evolutionary genetics analysis software. Bioinformatics.

[B46] Abe T (1949). Taxonomic studies on the puffers (Tetraodontidae, Teleostei) from Japan and adjacent regions – V. Synopsis of the puffers from Japan and adjacent regions. Bull Biogeogr Soc Jpn.

[B47] Miyadi D, Kawanabe H, Mizuno N (1976). Colored Illustrations of the Freshwater Fishes of Japan.

[B48] Society for nature conservation research of Fukui prefecture (1998). Animals of inland waters of Fukui prefecture.

